# White Matter Lesion Assessment in Patients with Cognitive Impairment and Healthy Controls: Reliability Comparisons between Visual Rating, a Manual, and an Automatic Volumetrical MRI Method—The Gothenburg MCI Study

**DOI:** 10.1155/2013/198471

**Published:** 2013-01-16

**Authors:** Erik Olsson, Niklas Klasson, Josef Berge, Carl Eckerström, Åke Edman, Helge Malmgren, Anders Wallin

**Affiliations:** ^1^Department of Neuroscience and Physiology, Institute of Psychiatry and Neurochemistry, The Sahlgrenska Academy, University of Gothenburg, 40530 Gothenburg, Sweden; ^2^Department of Philosophy, Linguistics and Theory of Science, University of Gothenburg, 40530 Gothenburg, Sweden

## Abstract

Age-related white matter lesions (WML) are a risk factor for stroke, cognitive decline, and dementia. Different requirements are imposed on methods for the assessment of WML in clinical settings and for research purposes, but reliability analysis is of major importance. In this study, WML assessment with three different methods was evaluated. In the Gothenburg mild cognitive impairment study, MRI scans from 152 participants were used to assess WML with the Fazekas visual rating scale on T2 images, a manual volumetric method on FLAIR images, and FreeSurfer volumetry on T1 images. Reliability was acceptable for all three methods. For low WML volumes (2/3 of the patients), reliability was overall lower and nonsignificant for the manual volumetric method. Unreliability in the assessment of patients with low WML with manual volumetry may mainly be due to intensity variation in the FLAIR sequence used; hence, intensity standardization and normalization methods must be used for more accurate assessments. The FreeSurfer segmentations resulted in smaller WML volumes than the volumes acquired with the manual method and showed deviations from visible hypointensities in the T1 images, which quite likely reduces validity.

## 1. Introduction

Age-related white matter lesions (WML) mainly affect information processing speed and executive function [1] and entail an increased risk for cognitive decline and disability [2]. In a meta-analytic study, high hazard ratios were also reported for incident stroke (3.3) and dementia (1.9) [3], but results from studies on WML association to dementia subtypes are inconclusive [4–6]. The prevalence of WML increases with age, and in a population study, 51% of randomly selected healthy subjects aged 44–48 had WML [7]. For the age range 60–64, all had WML, and 49% of these had at least one large (>12 mm) WML region [8].

In magnetic resonance (MR) imaging, white matter hypointensities in T1 weighted images, and white matter hyperintensities in T2 weighted and FLAIR images are regarded as visualizations of WML. The conditions for demarcation of WML are enhanced in FLAIR images due to suppression of the signal from free fluid. In contrast to T2 and T1 weighted images, this suppression causes fluid filled cavities in FLAIR images to be hypointensive and excluded from WML by the intensity definition. However, the intensity suppression in the FLAIR sequence entails incident imaging artifacts in the border region between WML and free fluid, for example, in the periventricular region [9]. WML visible in MR imaging reflect demyelinization, axonal loss, gliosis, or edema. However, WML MR imaging comprises no lucid separation between these pathological substrates.

It is customary to use CT or MR imaging of WML among the diagnostic criteria for subcortical vascular dementia, for example, in Erkinjuntti et al. [10]. However, standardization of WML estimation is needed in order to establish uniform diagnostic criteria in clinical practice. Visual rating is simple and fast and therefore an important candidate method for standardized clinical use. There are several visual rating methods for WML research purposes [11, 12]. Among these, the Fazekas visual rating [13, 14] is frequently used in research, and it has been shown to have good reliability compared to two other rating scales [12], but the results on its correlation to volumetrical assessment diverge. For example, the Fazekas visual rating has shown the highest [11] and lowest [15] correlation to volumetrical assessment compared to other visual rating scales. Manual volumetrical assessments have shown higher reliability than visual rating scales [11, 15] and would be valuable in clinical settings if made less labour intensive. In Gouw et al. [16], visual rating of WML and WML volumetry had similar correlations to neuropsychological performance, but in Garrett et al. [17], a correlation to neuropsychological performance was only found using WML volumetry and not in visual WML rating. Several segmentation and thresholding techniques have been used to manually assess WML volume in the literature, but one of the few techniques with a methodological description is reported in Gurol et al. [18].

Several automatic volumetry methods have been developed for WML classification [19–21]. FreeSurfer is one of these methods, and it contains automatic assessment of neuroanatomical subregions as well as WML hypointensity volumes. FreeSurfer has been frequently used for neuroanatomical subregional volumetry and has been shown to be comparable in accuracy to manual labeling for many tasks [22, 23] and to perform well compared to other automated segmentation tools [24]. However, few publications have reported FreeSurfer WML volumes at all, and only one study reports intermethod reliability figures [25]. The latter study also reported that regional WML predicted executive dysfunction. One study found that total WML was significant for AD versus controls [26], and another found that total WML predicted functional decline almost as well as the best predicting regional WML regions [5].

Reliability analysis is the first step in assessing the accuracy of a WML method, and the often reported excellent or near excellent reliability may be a reason why methodological issues about reliability have not always been given enough attention. The aim of the present study was to compare three types of assessment methods of total WML in a clinical sample in order to examine different aspects of their reliability and to determine features in need of further methodological development. The Fazekas rating was included in this study because of its simplicity. The manual MRIcron WML volumetry method was included on the basis of a presumed superiority in accuracy. FreeSurfer WML volumetry was included because of the automatic assessment of WML as well as the need for further validation because of the common use of the method.

## 2. Method

In the Gothenburg mild cognitive impairment (MCI) study, subjects between 40 and 86 years of age (mean 65.6, SD 7.7 years) with subjective or objective cognitive impairment were recruited from the Memory Clinic at the Sahlgrenska University Hospital. The selection process has been described in more detail by Eckerström et al. [27]. Exclusion criteria were acute somatic disease, severe psychiatric disorder, pseudodementia, and substance abuse or confusion caused by drugs. Controls were recruited from other medical studies and senior citizen organizations, and baseline exclusion criteria were subjective or objective signs of cognitive disorder. The study was approved by the Ethics Committee of the University of Gothenburg, and the subjects gave their informed consent to participate in the study.

The study subjects were biannually assessed and classified according to the Global Deterioration Scale (GDS) [28]. Subjects with a GDS 2 or GDS 3 score received an MCI classification. MCI subjects remaining in the GDS 2 to GDS 3 range at followup were classified as “MCI stable,” while MCI subjects receiving a GDS 4 (mild dementia) or higher classification at followup were classified as “MCI converting.” Twenty eight controls, 69 MCI stable, 9 MCI converting, and 46 dementia subjects were included in the present study. WML was measured on the first 1.5T MR imaging acquisitions in the study, between the years 2005 and 2007.

Imaging was performed on a Siemens Symphony 1.5T scanner. Axial T2 weighted images were used for the Fazekas rating, coronal FLAIR images were manually segmented in the MRIcron software, and coronal T1 weighted images were analyzed with the FreeSurfer package (stable release version 4.0.5). In Table 1, the scan parameters used in the study are presented. All raters and operators were blinded for header data like identity and cognitive status of the subject. This study report was outlined to be in accordance with the guidelines for reliability studies in Kottner et al. [29].

### 2.1. Visual Rating

WML severity was rated using modifications of the Fazekas scale [13]. Periventricular WML and deep WML were not rated separately, in accordance with the recommendations by Fazekas et al. [30], but only three grades were used as in Inzitari et al. [14] in contrast to the original four-graded Fazekas scale, and in the present study, grade 1 also included possible absence of WML. For each subject, the slice with the largest WML occurrence visible was used to determine the WML load as belonging to one of three grades. The assessments were performed independently by two raters (J. Berge and J. Eldblom) who compared the images with the template images in axial orientation of the different grades of WML given by [14]. Metrical measurements were not used. Only cerebral WML were included in the ratings. The raters J. Berge and J. Eldblom had substantial knowledge of neuroanatomy and went through limited training in visual rating before entering the study assessment.

### 2.2. MRIcron

Cerebral WML segmentation and intensity thresholding were performed on 1.5T coronal 5 mm FLAIR images using the MRIcron software with a modification (see later) of the method used in Gurol et al. [18]. The segmentation method involves an initial rough manual demarcation of WML, separating them from noncerebral regions and septum pellucidum, as in Holland et al. [31], within the same intensity span, followed by an intensity threshold set to separate WML from adjacent tissue types. In the Gothenburg MCI cohort, artifactual image intensity differences between slices and between series (using the same FLAIR sequence) were common. No automatic intensity normalization between patients was done, but in order to consistently analyze each patient's series in similar intensity settings, the MRIcron grayscale mapping was used on a window setting containing all brain tissue in a certain slice (containing the quadrigeminal plate). Due to intensity inhomogeneities, the manual volumetry method adapted from Gurol et al. [18] required a modification towards a more WML-specific manual segmentation, where the contour of the WML was demarcated quite closely. One rater (E. Olsson) demarcated 152 subjects, while a second rater (J. Berge) independently demarcated 27 randomly selected subjects for the determination of interrater reliability. The rater E. Olsson has longstanding experience in MRI segmentation, and the rater J. Berge went through substantial training in the manual WML volumetrical method before entering the study assessment.

### 2.3. FreeSurfer

Hypointensity volumetrics determined as WML were estimated by an operator (N. Klasson) running the FreeSurfer analysis (stable release version 4.0.5). FreeSurfer is a highly automatic image analysis suite and is available for download online (http://surfer.nmr.mgh.harvard.edu/). FreeSurfer uses a probabilistic atlas generated from manually segmented MR scans to execute the segmentation. The probabilistic information has been mapped into Talairach space so that each location therein contains specific probabilities for each tissue type. Given a specific location and tissue type, the probabilities are given as (1) a Gaussian intensity distribution, (2) probability of occurrence, and (3) probability of neighboring tissue types. During segmentation, a number of processes take place including motion correction and intensity normalization, removal of nonbrain tissue, Talairach registration, and labeling of voxels into tissue types. An initial labeling takes place where each voxel is assigned its most probable tissue type. An iterative algorithm then uses the found tissue probabilities to calculate new probabilities for the voxel labels. This process continues until no changes of tissue types take place. Manual edits were done by the operator (N. Klasson) to reduce inaccuracies in white and grey matter classification. The segmentation process has been described in detail elsewhere [22, 23].

### 2.4. Statistical Methods

The distribution of WML volumes in the Gothenburg MCI study was right skewed, and normality could not be assumed, not even after log transformation. The statistical analyses were done in SPSS 19. The reliability analyses comprised Spearman correlation, intraclass correlation (ICC) (with two-way mixed, absolute agreement and single measurements model), and Kendall's tau. Tertile selection was performed on the manual MRIcron volumetry distribution. Differences in reliability between the highest WML tertile and the lower tertiles were tested with Fischer's r to z transformation. For differences between raters and methods, Wilcoxon matched pairs test and Bland-Altman plots were used.

## 3. Results

### 3.1. Study Characteristics

The study characteristics for the controls, MCI stable, and dementia groups are presented in Table 2. Dementia patients had significantly lower MMSE scores than all other groups, had significantly lower education than stable MCI, and were significantly older than stable MCI. All three WML assessment methods detected significant associations between WML estimate and age (Fazekas 0.385, manual MRIcron 0.334, automatic FreeSurfer 0.524).

### 3.2. Reliability

**Figure 1:**
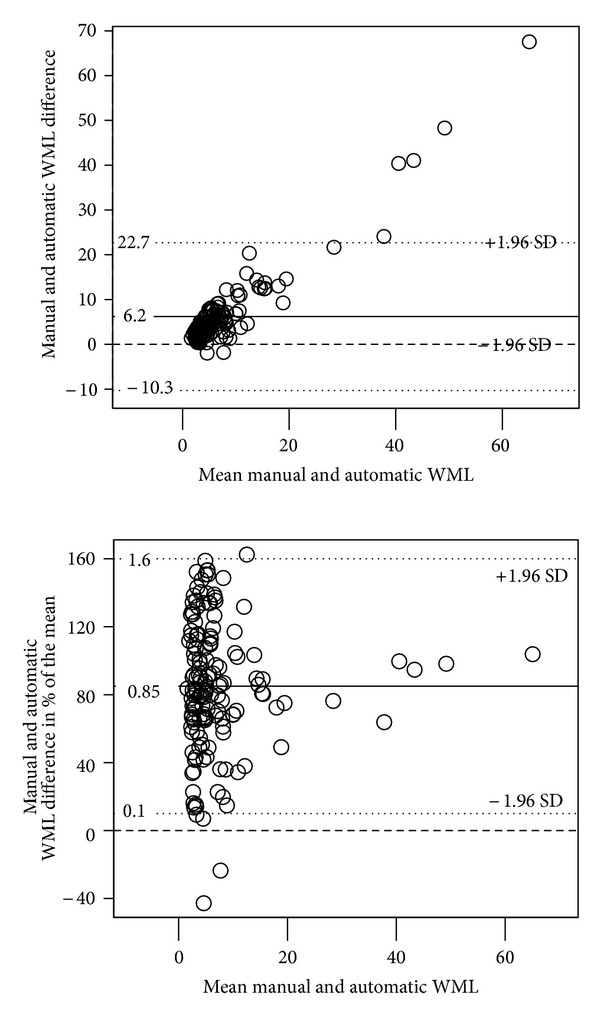
Bland-Altman plots of the intermethod differences (manual MRIcron minus automatic FreeSurfer WML volumes in cm^3^). The *x*-axes contain mean WML volumes; the *y*-axis contains absolute differences in (a) and fractional differences of mean volume in (b). The dashed line designates the no differences level.

Regarding interrater reliability, the Spearman rho coefficient was 0.89 with regard to the modified Fazekas visual rating scale and 0.60 regarding the manual MRIcron WML volumetry. The rho value was 0.65 for the intermethod correlation between FreeSurfer automatic WML volumetry and manual WML volumetry (Table 3). No significant systematic differences between raters were found for Fazekas visual rating or for MRIcron manual volumetry in the Wilcoxon matched pairs test (details not shown), but the FreeSurfer automatic volumetry differed significantly from the manual MRIcron volumetry. Almost all FreeSurfer volumes were lower than the corresponding manual MRIcron volume (Figure 1).

**Figure 2:**
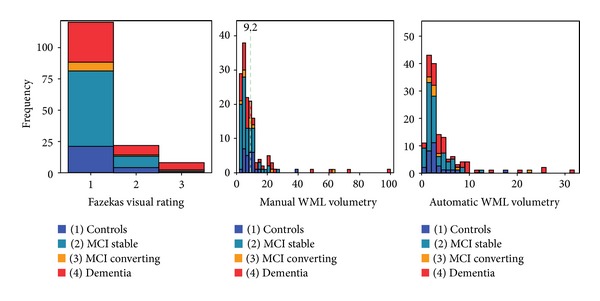
Histograms of WML distribution in the three methods compared. (a) Fazekas visual rating (grade). (b) Manual MRIcron volumetry (cm^3^). Dashed green line shows tertile 2 cut off. (c) Automatic FreeSurfer volumetry (cm^3^).

In order to evaluate reliabilities in the dense and the sparse parts of the data distribution (Figure 2), respectively, the WML volumes were separated into tertiles (Table 3). For the aggregated lower two tertiles, the Fazekas interrater reliability (Spearman's rho) measured 0.65, and for the upper WML tertile 0.92, this difference was significant. The manual MRIcron interrater reliability was nonsignificant for the lower two tertiles (which make up about 10 percent of the whole volume range) but significant for the upper WML tertile with an interrater reliability of 0.94. For the anterior part of the brain taken separately, there was still no significant reliability for the lower aggregated WML tertiles, but it was significant for the posterior part with a rho value of 0.56. For the intermethod reliability between the manual MRIcron volumetry and the automatic FreeSurfer volumetry, the rho value was also lower, 0.38 in the lower aggregated tertiles, than in the upper tertiles, 0.74. 

For ICC values, see Table 3. The reason why we do not report them in the text is stated in Section 4.

**Figure 3:**
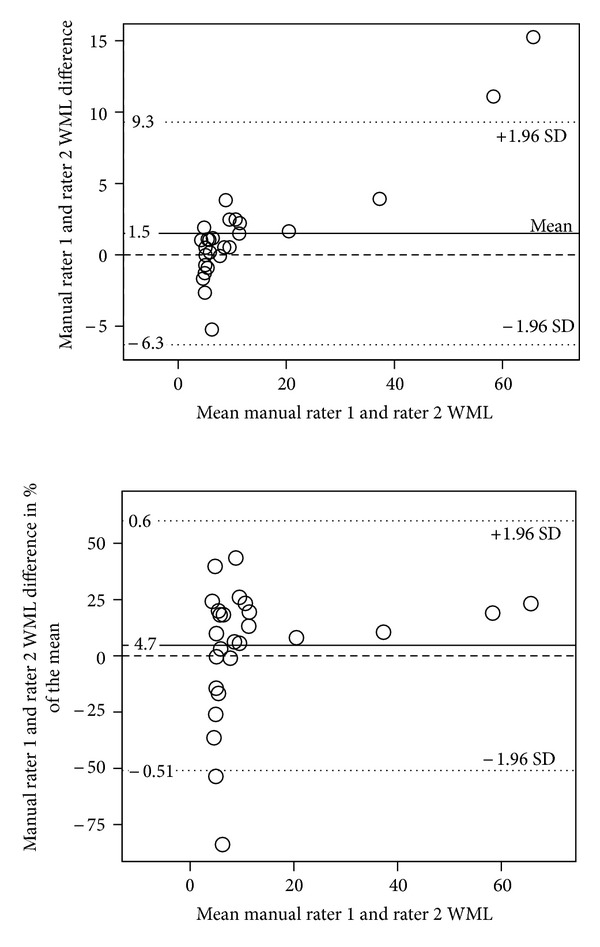
(a) and (b) Bland-Altman plots of the interrater differences for the manual MRIcron WML method in cm^3^. The *x*-axes contain mean WML volumes; the *y*-axis contains absolute differences in (a) image and fractional difference of mean volume in (b). The dashed line designates the no differences level.

Figure 3(a) shows the Bland-Altman (BA) plots of absolute volume interrater differences in the manual MRIcron method, demonstrating increasing differences with higher volumes. However, the variation in rater differences was larger in the assessments of the lowest range of the WML volumes embracing about 80% of the subjects. When comparing the difference as a percentage of the mean measures of each subject (Figure 3(b)), the variation was even more pronounced with regard to the lowest part of the WML volumes, while there was no clear indication of an increase in rater differences with larger volumes.

The intermethod BA plots (Figure 1) for the manual MRIcron volumetry and automatic FreeSurfer volumetry showed a similar pattern as the interrater BA plots, with a pronounced variation for low WML volumes in Figure 1(b).

## 4. Discussion

### 4.1. Reliability

Conventional interrater reliabilities were acceptable for the Fazekas rating and the manual MRIcron volumetry. The intermethod reliability for the manual MRIcron and automatic FreeSurfer methods was also acceptable. It is common to evaluate reliability in WML research with intraclass correlation (ICC) with excellent results, for example, in Gao et al. and Smith et al. [11, 25]. However, it is misleading to use such an analysis in a data structure where the distribution is skewed, with very sparse data points in the upper third of the volume range. While showing an excellent reliability for the whole sample, like previous studies, the ICC in the lower aggregated tertiles for the manual method was nonsignificant (Table 3). The density of low-burden WML (Figure 2) probably represents a very common clinical distribution, and reliability analysis must under these conditions be performed and interpreted with caution.

The finding in the present study of lower reliability in the Fazekas rating with lower WML burden is congruent with the finding by Wardlaw et al. [32] where cohorts with lower WML burden showed lower reliability. The higher reliability for high WML burden has been considered as a ceiling effect, but there might as well be floor effects in visual rating [33]. Presumed ceiling and floor effects do not disqualify visual rating as a candidate for clinical use, but such effects would in general lower the usefulness for WML research, for example, the possibility to find valid correlations with psychometrics.

The nonsignificant Spearman correlation in the aggregated lower tertiles between manual MRIcron volumetry raters could possibly be due to the high density of data affecting the rank order of the ratings. It is unclear if the high Spearman correlation for high-burden WML implies an overestimation of the reliability due to the sparse data density (leading to less error with ranked data) or if there really was a higher reliability as the BA plot of fractional differences seems to imply (Figure 3(b)). In our opinion, the low reliability in low-burden WML is most probably not due to the combination of high density of data and the nature of the Spearman correlation. Rather, it may be a real rater- and intermethod variation as is visible in the BA plots which seem to indicate an increased variation in the lowest quarter of the volume range. The higher rater variation in low WML may in turn be due to difficulties in the handling of intensity distortion affecting the thresholding step in the manual volumetry.

**Figure 4:**
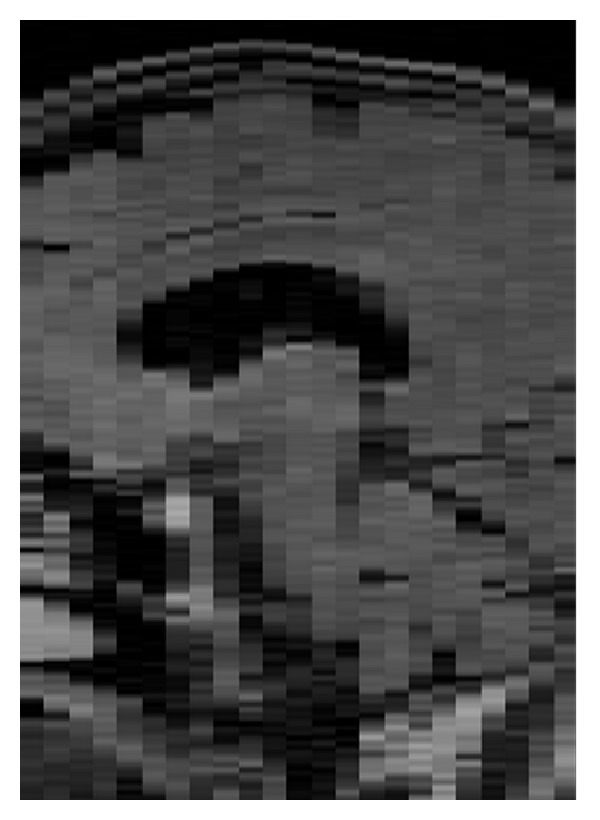
Sagittally reformatted slice from an example subject showing the common anterior intensity shift between the fourth and fifth slices in the coronal 2D FLAIR sequence.

Intensity inhomogeneities in MR images and variation in grayscale level between scan series result in inconsistent classifications of hyperintensities that in turn introduce measurement errors in the manual WML assessment. In order to assess WML volumes as accurately as possible under conditions with varying intensity levels through the image slices of a subject, a methodology was chosen where the thresholding was adjusted as a compromise to best fit the visible WML volume through all image slices of a subject's brain. Since the localization and extent of WML vary, the accuracy of the thresholding can be expected to be decreased by these intensity distortions. A particular shift in grayscale level was observed in the anterior part of all FLAIR image series (Figure 4), which may be due to gradient eddy currents or cross talk between 2D FLAIR slices [34, 35]. The unreliability for low WML seems to emanate mainly from the anterior half of the image data, and the intensity shift in the anterior part may well be a reason for more complex considerations in the thresholding of low WML burden cases. For higher WML volumes, the thresholding step in general only affects the amount of WML selected, but for lower volumes, the thresholding more often affected the presence or absence of WML in a slice, which means a more straightforward thresholding with higher volumes. In short, increased complexity in the thresholding may be the main cause of an increased rater variation for low WML volumes.

**Figure 5:**
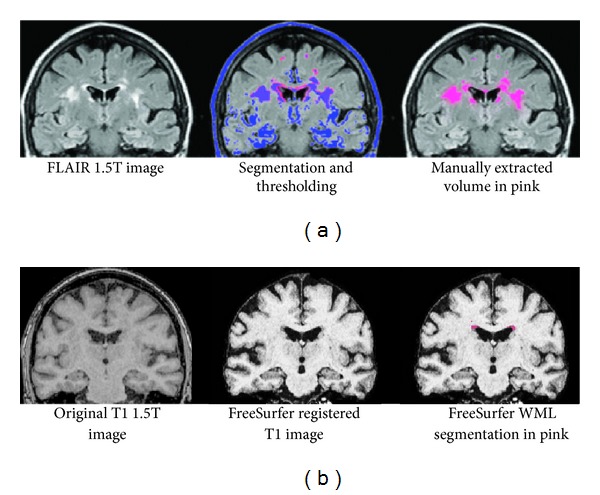
1.5T MR images from a patient with severe-burden WML showing the differences in scan sequence and WML detection between manual MRIcron volumetry (a) and automatic FreeSurfer volumetry (b).

**Figure 6:**
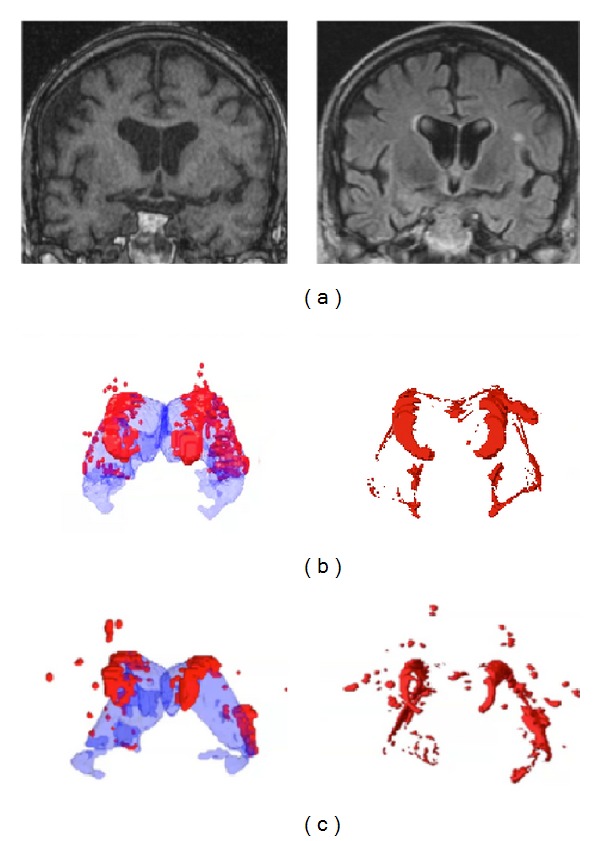
3D visualizations and MRI images of WML assessed by FreeSurfer and in manual volumetry. (a) MRI images of a dementia patient. The left image shows a T1 original image used in the FreeSurfer assessment where there are FreeSurfer findings of punctate WML. The FLAIR image to the right in the corresponding position contains a large punctate WML patch close to the superior cortical part of the left insula which is hardly visible in the T1 image. (b) The same dementia patient with WML burden (in red) at approximately the same manually extracted high-burden WML volume as the control subject. FreeSurfer assessment is shown to the left with the lateral ventricles visualized in transparent blue, and manual volumetry is shown to the right. (c) Control subject with WML burden (in red) at approximately the same manually extracted high-burden WML volume as the dementia patient. FreeSurfer assessment is shown to the left with the lateral ventricles visualized in transparent blue. Manual volumetry is shown to the right.

The FreeSurfer suite includes intensity normalization steps which to some extent will limit intensity distortion, but that also may extinguish some of the hypointensities. Further, the T1 MR sequence used for the FreeSurfer volumetry does in general have lower WML definition and somewhat smaller areas of WML hypointensities compared to the hyperintensities in the FLAIR sequence. A visual inspection of the T1 weighted images and the FreeSurfer segmentations confirmed that the substantial deviations between the volumetrical methods in the detection of WML in various locations may mainly be due to the lower definition in the T1 weighted images. The FreeSurfer segmentation often omitted large amounts of deep WML seen in the FLAIR images (Figure 5). On few occasions, FreeSurfer classified sulcal cortical voxels as WML. Occasionally, FreeSurfer detected more WML in the periventricular region than visible in the T1 weighted images. Compared to WML seen in FLAIR images, the amount of WML found by FreeSurfer still was lower even in the periventricular regions. The T1 MRI images in Figures 5 and 6 are examples showing that the noise level makes the detection of punctate WML a difficult task, nevertheless FreeSurfer detects several punctate WML patches in this region. The single punctate WML patch in the FLAIR slice in Figure 6 is hardly visible in the T1 slice, and it is conceivable that no punctuate WML patch in this location is detected in FreeSurfer. In some cases, FreeSurfer detected small punctate WML not detected in the manual volumetry (Figure 6(b)), but occasionally, it detected less punctate WML than the manual volumetry (Figure 6(c)). Hence, FreeSurfer seems to detect punctate WML in a largely random way. However, a more accurate punctate WML detection may be a disadvantage in dementia research since punctate WML often originate from perivascular spaces and have been found to have low progression [36]. In short, the FreeSurfer volumetry generally detects less WML, which is likely due to a combination of the method, the characteristics of WML, and the visibility of WML in T1 weighted images and leads to larger differences between the methods when measuring larger WML volumes (Figure 1).

WML progression generally begins in the periventricular region and subsequently expands radially to more peripheral locations [37]. The skewness of the distribution of WML, with a low fraction of high-burden WML subjects also in the dementia part of the sample, can be expected to exist also for clinical samples in general. The statistical atlas used in FreeSurfer was generated from a small sample (http://surfer.nmr.mgh.harvard.edu/fswiki/Buckner40Testing), where high-burden WML cases might be rare or lacking. An atlas generated with a low frequency of high-burden exposure may contribute to the FreeSurfer inability to find deep WML.

### 4.2. Limitations

The Fazekas rating was performed by comparing the largest WML aggregation to the templates, and no metrical measurements were used which possibly affected the classification of the subjects close to the Fazekas definitions of the metrical borders. The visual rating in three grades differs from the design in other studies, which limits the comparability of results. It is not feasible to use a zero grade WML in T2 images with any accuracy, and in a later control of the FLAIR images, it was ascertained that no subjects with complete absence of WML were present in the study. In visual rating, the FLAIR sequence can be expected to be advantageous compared to the T2 sequence due to the signal suppression of free fluid in FLAIR images. However, the FLAIR sequence in this study had coronal orientation and 5 mm slice thickness, which made it unsuitable to use with the axial template images. Therefore, the axial T2 scan sequence was used.

In the manual method, the basal ganglia nuclei in FLAIR images often had no clear intensity separation from white matter, which may have contributed to an inaccurate volume assessment in this region. The hyperintensities along the ventricular lining in the basal ganglia region were included in the manually assessed WML, which may be unsuitable and possibly confound the differentiation, primarily in patients with low WML volumes. The different scan sequences used in the different methods confound the methodological comparison; for example, only the FLAIR sequence does to some extent show separate intensity ranges for fluid filled cavities and WML. By contrast, in T1 and T2 sequences, fluid-filled cavities have intensities in the same range as WML.

The moderate interrater correlation for the manual volumetry could possibly be a consequence of intensity distortions in the FLAIR images. Although steps were taken in the thresholding methodology to minimize the impact of intensity distortions, sample tests of WML volume using different thresholdings unveiled considerable variation in measurements. The FLAIR sequence had obvious intensity inhomogeneities, and no automatic intensity normalization was performed which may limit the possibilities to interpret the present findings further.

## 5. Conclusions 

Reliability analysis showed acceptable overall results but with lower reliability for all methods in the lower aggregated tertiles. Despite excellent overall reliability for manual volumetry, no significant reliability was found in the lower aggregated tertiles. Hence, the results of intraclass correlation in WML samples, that is, commonly skewed, might be misleading, and reliability analysis of WML methods should be considered with caution. Image intensity distortion seems to be the major cause of the reliability deficits. Optimized MR imaging, postscan intensity standardization and normalization are quite likely among the most important factors to consider for more accurate measurements of WML. FreeSurfer comprised lower volumes than the manual method, probably due to the T1 sequence it uses, and was not able to detect punctuate WML in a consistent manner.

### 5.1. Future Directions

A medically oriented follow-up paper focusing on the predictive power of the different WML measurements is being prepared. In another study, we intend to refine the visual comparison between the methods, using a larger sample of subjects and coregistration of images from different scan series. Finally, our long-term goal is an improved manual method that excludes thresholding but includes an adequate intensity normalization.

## Figures and Tables

**Table 1:** Scan parameters 1.5T Siemens Symphony (Syngo MR 2004A 4VA25A).

		2D FLAIR	T2 2D TSE	T1 3D MPR
		Coronal	Axial	Coronal
Repetition time	ms	8780	5410	1610
Echo time	ms	112	104	2.38
Number of averages		1	2	1
Flip angle	°	150	150	15
Slice thickness	mm	5	5	1
Spacing between slices	mm	5.5	6	1
Acquisition pixel size	mm	1.3∗0.9	0.9∗0.4	1.0∗1.0
Reconstruction pixel size	mm	0.45∗0.45	0.45∗0.45	0.49 ∗ 0.49
Matrix size		512∗416	512∗416	512∗416
Field of view	mm	230∗187	230∗187	250∗203

**Table 2:** Study characteristics. *P*-values were assessed by the two-tailed *t*-test except gender where chi square was used. Gr 4 is group 4 Dementia. Gr 2 and MCI-s are stable MCI. MCI-c is converting MCI. Gr 2 and Gr 4 had significantly lower scores than the group specified in the cell for the pertaining variable; for example, Gr 2 (stable MCI) had significantly lower age than group 4 (dementia group). There was no significant difference in gender.

	1 Controls		2 MCI-s		3 MCI-c		4 Dementia		Gr 2	Gr 4
	Mean	SD	Mean	SD	Mean	SD	Mean	SD	*P* < 0.05	*P* < 0.05
Age	66.5	8.2	63.9	7.2	65.6	7.2	67.6	8.0	4	
Education years	11.7	2.3	11.8	3.4	12.6	4.5	10.4	3.7		2
MMSE	29.3	0.4	28.3	1.9	28.4	1.7	25.3	2.9		1, 2, 3

	Count		Count		Count		Count			

Gender M/F	9/19		31/38		6/3		21/25		ns	

**Table 3:** Inter-rater and inter-method reliability. *Correlation was significant at the 0.05 level (2-tailed). **Correlation was significant at the 0.01 level (2-tailed). ManWML is manual MRIcron volumetry; *n* = 27. Anterior is the manual MRIcron assessed volume anterior of the quadrigeminal plate. Posterior is the manual MRIcron assessed volume posterior of the quadrigeminal plate. ManWML versus AutoWML is the manual MRIcron volumetry correlation to the automatic FreeSurfer volumetry. Rho below T3 is the Spearman correlation, and ICC below T3 is the intraclass correlation in the lower two tertiles. Rho in T3 is the Spearman correlation and ICC in T3 the intraclass correlation in the highest tertile. The reliability was significantly higher in the highest tertile for all methods.

	Kendall's tau-b	ICC	ICC below T3	ICC in T3	Rho	Rho below T3	Rho in T3
Inter-rater reliability							

Fazekas	0.88**	0.87**	0.65**	0.86**	0.89**	0.65**	0.92**
ManWML	0.45*	0.97**	0.19	0.95**	0.60**	−0.03	0.94**
Anterior					0.63**	0.23	0.94**
Posterior					0.82**	0.56*	1.00**

Inter-method reliability							

ManWML versus AutoWML	0.48**	0.51**	0.14**	0.44**	0.65**	0.38**	0.74**
